# How I Treat *TP53*-Mutated Acute Myeloid Leukemia and Myelodysplastic Syndromes

**DOI:** 10.3390/cancers14184519

**Published:** 2022-09-18

**Authors:** Michael Loschi, Pierre Fenaux, Thomas Cluzeau

**Affiliations:** 1 Hematology Department, University Hospital of Nice, Cote d’Azur University, 06202 Nice, France; 2 INSERM U1065, Mediterranean Center of Molecular Medicine, 06200 Nice, France; 3 Senior Hematology Department, Saint Louis Hospital, 75010 Paris, France

**Keywords:** AML, *TP53*, magrolimab, eprenetapopt, allogeneic stem cell transplantation

## Abstract

**Simple Summary:**

*TP53* mutations are seen in 5–10% of de novo MDS and AML, but 25–40% of therapy-related MDS and AML. Despite the addition of recent drugs to the common regimen and improvement of post transplantation survival, these particular myeloid malignancy subtypes remain a challenge for hematologists around the globe. In this article, we aim at reviewing the biology and most recent advances in the treatment of *TP53* MDS and AML.

**Abstract:**

*TP53*-mutated acute myeloid leukemia (AML) and myelodysplastic syndromes (MDS) are among the myeloid malignancies with the poorest prognosis. In this review, we analyze the prognosis of these two diseases, focussing particularly on the extent of the mono or biallelic mutation status of *TP53* mutation, which is largely correlated with cytogenetic complexity. We discuss the possible/potential improvement in outcome based on recent results obtained with new drugs (especially eprenetapopt and magrolimab). We also focus on the impact of allogeneic hematopoietic stem cell transplantation (aHSCT) including post aHSCT treatment.

## 1. Introduction

Tumor protein p53 (*TP53*), a tumor suppressor gene, is the most frequently mutated gene in cancer [1]. *TP53* is the “guardian of the genome” via several functions such as regulation of metabolic functions, apoptotic pathways, cellular senescence, and DNA repair [2]. Myelodysplastic Syndromes (MDS) and Acute Myeloid Leukemia (AML) with *TP53* mutation generally have poor prognosis, almost irrespective of the treatment administered, including after allogeneic Hematopoietic Stem Cell Transplantation (aHSCT). Due to their specific characteristics, experts in the fields have recently argued that *TP53*-mutated AML/MDS require special consideration [3,4,5].

## 2. Landscape of *TP53* Mutations

An analysis of the *TP53* whole coding sequence found that about 85% of mutations were clustered between codons 125 and 300, which is the location of the DNA-binding domain. The mutation types are mostly missense (90%). Outside this region, the majority of mutations are nonsense or frameshift. 

Hotspot mutations are localized in the DNA-binding domain and R175H is one of the most frequent hotspot mutants [6]. The majority of *TP53* mutations have an impact on protein structure and are classified as “contact” (R248 and R273) or “structural” (R175, G245, R249, and R282) [7]. These mutations are associated with a loss of function. 

In its target genes p53 also modulate transcription by interfering with so called response elements (REs) in the promoters or introns [8]. As a consequence, a loss of function has been reported in all the mutants. 

Finally, several hotspot mutants have been shown to impact cellular transformation [9], defining gain-of-function (GOF) mutations. Mutant p53 proteins may also exert dominant–negative effects (DNE) over wild-type p53.

## 3. *TP53* Mutations in AML and MDS

*TP53* mutations are seen in 5–10% of de novo MDS and AML patients, 25–40% of therapy-related MDS and AML patients [10,11], and 50% of patients with a complex karyotype [12]. Complex karyotypes are usually monosomal, very frequently include 5q deletion, and generally include 17p deletion, the latter being sometimes difficult to detect in complex cytogenetic rearrangements. They largely predominate in elderly patients [13]. In these patients, *TP53* mutation is generally biallelic, with the loss of one allele and the mutation of the remaining one. *TP53* mutation is however also seen in about 20% of low risk MDS patients with 5q deletion, where it is generally monoallelic. This MDS subset predominates in females, and the median age is lower than that of AML or MDS with a complex karyotype and biallelic *TP53* mutation. 

As in other cancers, the majority of *TP53* mutations in AML and MDS, are missense and localized in the DNA-binding domain [14]. It is unclear if the deleterious effect is only due to loss of function (especially in case of biallelic mutation) [15], or could include dominant negative effect or gain of function. *TP53* mutations usually confer a poor prognosis in MDS and AML [16]. This is especially the case for biallelic mutations, generally corresponding, as mentioned above, to mutations of one allele and loss of the other through 17 deletion, in complex monosomal karyotypes. Monoallelic mutations, generally corresponding to point mutation have less impact on outcome. Nevertheless, in the case of MDS with no excess of blasts, where it occurs in 20–25% of the cases, monoallelic *TP53* mutation is associated with a poorer response to treatment and survival [13,17]. 

## 4. Treatment of AML and MDS with *TP53* Mutation by Intensive Chemotherapy

In AML, eligibility for classical intensive chemotherapy (IC), generally with an anthracycline and cytosine arabinoside (AraC) [16], is mainly based on age and comorbidities. However, it also includes disease characteristics such as cytogenetic complexity and complex karyotypes, especially monosomal karyotypes, which are associated with poorer complete response (CR) rates, and very short responses [18,19,20,21,22,23,24]. 

In patients with a complex karyotype, those with *TP53* mutations appear to respond particularly poorly. Their outcome is not improved, contrary to some other types of AML, by the addition of the CD33 inhibitor gemtuzumab [25,26], by the use of encapsulated anthracycline-AraC molecules (CPX 351), the addition of lomustine, or by maintenance treatment with CC486 [21,22,25,27,28,29,30,31,32,33]. In *TP53*-mutated AML patients who achieve CR with IC, allo SCT is therefore recommended whenever possible, but is associated with a high relapse risk in this situation, as shown below (Table 1).

MDS, in general, are not treated with IC, with the exception of some candidates to allo SCT, in order to reduce the blast infiltration before transplant. Response to IC is mainly observed in the absence of a complex karyotype, which most *TP53*-mutated patients carry, as described above.

## 5. Hypomethylating Agents (HMA) Alone

A HMA alone has been used in the treatment of AML until recently and is now considered unfit for IC (based on age, comorbidities and/or the presence of a complex karyotype), while in MDS it remains the reference treatment in most higher risk cases. In AML, Azacitidine (AZA) yields response rates of 30 to 35% including about 30% of CR/CR with incomplete blood count recovery (CRi) [34], Decitabine (DAC) response rates of about 20% including 13% CR/CRi [28], and both drugs yield a median overall survival (OS) of about 10.5 months, which may be better than that obtained with low dose cytarabine, especially in patients with a complex karyotype. In higher risk MDS, median OS with AZA or DAC ranges from 18 to 24 months [35]. 

In *TP53*-mutated AML patients, AZA was shown to yield a 20–30% CR rate and a median OS at 7 months [36]; and DAC a 20–30% CR rate and a median OS of 6–12 months [37,38,39,40].

Using a 10-day regimen of DAC was shown to improve the outcome in one study, although this result was disputed [37].

Furthermore, TP53 mutation has an unfavorable impact on the outcome of higher-risk MDS treated with AZA, with lower response rates but, more importantly, shorter response duration and a median OS of 12.4 months [41] (Table 2).

## 6. HMA in Combination with Other Drugs in AML and MDS with *TP53* Mutation

The combination of AZA and Venetoclax is superior to AZA alone in AML in the elderly as a whole but also for patients with comorbidities who are not eligible for intensive treatment. It has become the reference treatment of AML in this group, and combinations with other agents have also been tested. However, in higher-risk MDS no combination has so far demonstrated a benefit over AZA or DAC alone in a phase 3 trial, but a recent phase 1/2 trial reported encouraging results on the combination of AZA and venetoclax [42] (Table 3).

### 6.1. HMA + Venetoclax

AZA combined with venetoclax (VEN) has become the standard of care for AML patients ineligible for IC, based on its superiority over AZA alone [43]. In *TP53*-mutated AML patients, while an increase of ORR was observed with HMA + VEN (55% vs. 0% in patients treated by AZA alone), responses were short with these combinations, and no significant OS benefit was observed (median OS was 6 months independent of treatment arm) [44,45,46]. In high-risk MDS patients, a recent phase 1/2 trial reported that the combination of AZA + VEN was safe, and demonstrated encouraging results in this group of patients [42]. Therefore, HMA + VEN may be a useful combination to bridge a patient to allo SCT, but is insufficient in itself. 

### 6.2. Azacitidine + Eprenetapopt

Eprenetapopt (APR-246) is a small molecule and its mechanism of action has not been completely elucidated. It has been shown to selectively induce apoptosis of *TP53*-mutant cancer cells. After conversion to methylene quinuclidinone (MQ), it covalently binds to mutant p53 to restore wild-type conformation, resulting in cell cycle arrest and apoptosis [50]. Aside from this mechanism, it may induce depletion in glutathione (GSH), increase oxidative stress [51,52,53,54], and induce ferroptosis [55]. Several preclinical and clinical studies have reported a synergistic effect with AZA [56].

These early observations have led to two clinical trials, conducted in the US and France, to assess the efficacy and safety of a combination of AZA + eprenetapopt in intermediate, high, and very-high IPSS-R risk myeloid malignancies (AML and MDS) (only 20 to 30% of blasts in the US trial) [47,48]. 

The US phase 2 trial enrolled fifty-five patients (40 MDS/11 AML) with a median age of 66 years. The Overall Response Rate (ORR) was 71% with a CR rate of 44%, with 38% undetectable *TP53* measurable residual disease (Next Generation Sequencing (NGS)) with *TP53* NGS negativity. Median CR duration was 7.3 months; with a median follow up of 10.5 months, median OS was 10.8 months. 

In the French phase 2 trial, fifty-two patients (34 MDS/18 AML) with a median age of 74 years were enrolled. The ORR was 58%, with a CR rate of 37% and an undetectable *TP53* measurable residual disease (MRD) of 30% (NGS). Median CR duration was 11.7 months; with a median follow up of 9.7 months, median OS was 12.1 months.

Regarding safety, no additional hematological toxicity was reported compared to azacitidine alone, however, neurological side effects including ataxia, cognitive impairment, acute confusion, isolated dizziness, and facial paresthesia were reported in 40% of patients (6% were grade 3 or 4, and all were fully reversible without recurrence after dose reduction). 

Patients with *TP53*-mutated MDS fared better than AML patients (for AML patients, a phase 1 study evaluating a combination of AZA + VEN + eprenetapopt in *TP53*-mutated AML is ongoing (clinicaltrials.gov NCT: NCT04214860)). 

Based on the results of these phase 2 trials, a phase 3 randomized clinical trial was conducted to compare AZA + eprenetapopt to AZA monotherapy in *TP53* MDS patients (clinicaltrials.gov NCT: NCT03745716). The results did not demonstrate superiority for the combination when compared to azacitidine alone. Despite these results, eprenetapopt is now being investigated in the transplant setting, especially to decrease the tumor burden before and after transplant by providing a significant reduction in MRD.

### 6.3. Azacitidine + Magrolimab

Magrolimab (MAGRO) is a first-in-class investigational monoclonal antibody against CD47 and a macrophage checkpoint inhibitor that is designed to interfere with the recognition of CD47 by the SIRPα receptor on macrophages, thus blocking the “don’t eat me” signal used by cancer cells to avoid being ingested by macrophages. Overexpression of CD47 is an adverse prognostic factor and could be a therapeutic antibody target on human acute myeloid leukemia stem cells. CD47 blockade induces tumor phagocytosis and eliminates leukemia stem cells in AML models [57]. AZA has been shown to both increase expression of CD47 and the pro-phagocytic signal calreticulin in myeloid malignancies. AZA synergizes with MAGRO by inducing “eat me” signals on AML, to enhance phagocytosis [58]. 

In a phase 1b trial of AZA + MAGRO in AML patients, where the MAGRO schedule was 30 mg/kg IV weekly or Q2W associated to AZA 75 mg/m²/d days 1–7 on 28-day cycles, results for 64 patients were reported. The ORR was 63% including 42% CR and 12% CRi. Forty-five percent of patients obtained a complete cytogenetic response (CCR) and 35% obtained MRD negativity. The median duration of response was 9.6 months.

The study population was enriched with *TP53*-mutated patients (29/43 (67%)), whose ORR was 69% including 45% CR and 14% CRi, with a median duration of response of 7.6 months, 44% of CCR, and 29% MRD negativity, and a median OS of 12.9 months (vs. 18.9 months in *TP53* wild-type patients) [49].

A phase 3 clinical trial evaluating AZA + VEN vs. AZA + MAGRO in *TP53*-mutated AML (clinicaltrials.gov NCT04778397) and a phase 1/2 study evaluating AZA + VEN + MAGRO (clinicaltrials.gov NCT04435691) are currently enrolling.

### 6.4. AZA+ Sabatolimab

Sabatolimab is a T-cell immunoglobulin and mucin domain-containing protein 3 (TIM3) inhibitor. TIM3 is a member of the TIM family, that was originally identified as a receptor expressed on T cells. Recent data have demonstrated that TIM3 works as a co-inhibitory receptor (checkpoint receptor), exhibited by dysfunctional or ‘exhausted’ T cells. Sabatolimab was tested in combination with HMAs in AML and higher-risk MDS. Preliminary results of AZA + sabatolimab trial, presented at the EHA (European Hematology Association) 2021 meeting suggested promising results in a limited number of *TP53*-mutated patients, with an ORR of 71.4% including 28.6% CR, and 14.3% marrow CR [59].

## 7. New Combinations Needing Investigation

Due to the high frequency of *TP53* mutations in cancer, and their resulting poor prognosis, many drugs are being tested in vitro for their ability to inhibit mutated p53, reconform it, and/or activate some of its targets. 

Drugs already used in other diseases may be particularly interesting to develop in *TP53*-mutated AML and MDS.

As an example, Chen et al. recently showed that Arsenic Trioxyde (ATO) rescues structural p53 mutations through a cryptic allosteric site [60]. 

Sixty percent of *TP53* mutations are conformational in MDS and AML and could be restored by ATO. The Groupe Francais des Myelodysplasies (GFM) obtained a 19% response rate with ATO in MDS. Roboz et al. [61] reported 34% CR in AML with the combination of low dose aracytine and ATO (including 30% in secondary or therapy related AML and 30% in patients with unfavorable cytogenetics). However, no analysis of *TP53* mutation was carried out in either study. Combinations of AZA + ATO (+/− VEN) warrant evaluation in AML and MDS with *TP53* mutation. 

Other drugs aiming specifically at mutated *TP53* are being developed. ReACp53, a cell-penetrating peptide, designed to inhibit p53 amyloid formation, has shown rescue p53 function in cancer cell lines and in organoids, and is in evaluation in some solid *TP53*-mutated cancers [62]. COTI-2, a novel thiosemicarbazone derivative, normalized wild-type p53 target gene expression and restored DNA-binding properties to the p53-mutant protein [63]. Niclosamide, an anthelmintic drug, inhibited ASAP2, a member of the ArfGAP family, which is overexpressed in pancreatic ductal adenocarcinoma characterized by four main driver genes KRAS, *TP53*, CDKN2A, and SMAD4. Niclosamide was able to bypass the effect of *TP53* mutations in other models and need to be explored [64]. Other drugs in development for solid cancers could be also interesting such as Ataxia Telangiectasia Mutated (ATM) inhibitors, ataxia telangiectasia mutated, and Rad3-related (ATR) inhibitors. The Rad3 gene is required for cell viability and excision repair of damaged DNA, and is also called the FRAP-related protein 1 (FRA 1). ATR is encoded in humans by the ATR gene. Furthermore, Wee1 inhibitors and Checkpoint kinase (CHK) inhibitors may be potential candidates [65,66,67]. Wee 1 kinase is gatekeeper of the G2-M cell cycle checkpoint that allows DNA repair before mitotic entry. Targeting Wee 1 for inhibition potentiates chemotherapy because Wee 1 is highly expressed and active in several cancer types that are dependent on a functional G2-M checkpoint for DNA repair. More recently, investigational bispecific dual-affinity retargeting antibody flotetuzumab was assessed in patients with relapsed refractory AML. Seven out of 15 patients achieved complete remission and a median overall survival of 10.3 months [68]. These results emphasized the central role of immunotherapy in this highly chemoresistant AML subtype.

## 8. Allogeneic Stem Cell Transplantation

Allogeneic stem cell transplantation (aHSCT) remains the only curative option for patients with *TP53*-mutated AML or MDS. However, the presence of the *TP53* mutation in AML or MDS is associated with a high risk of relapse pretransplant, and is the main cause of death following transplantation (Table 4). A retrospective analysis of 30 patients who underwent aHSCT for AML (n = 19) or MDS (n = 11) with chromosome 17 abnormalities, reported a poor outcome for patients with the *TP53* mutation [69]. Patients experienced short relapse-free survival, with a median relapse-free survival of 6- and 4-months post-transplant for the AML and MDS patients, respectively, (*p* > 0.5). The non-relapse mortality (NRM) was 10.6% (2/19 patients) and 9.1% (1/11 patients) for AML and MDS patients, respectively, (*p* > 0.5). The median overall survival post-transplant was 18 months for AML and 11 months for MDS patients respectively (*p* > 0.5). In a recent retrospective report from the European Society for Blood and Marrow Transplantation (EBMT), the relapse rate for patients with a 17p abnormality was as high as 61% at 2 years [70]. The subsequent 2 year OS and leukemia free survival (LFS) were 28 and 24%, respectively. The study enrolled 125 AML patients carrying a 17p abnormality who received an aHSCT in CR1. In multivariate analysis only, the presence of a −5/5q- in addition to abn (17p) was significantly and independently associated with worse OS, LFS, and higher relapse incidence. The type of conditioning was not significantly associated with the outcome. An American retrospective study by the Center for International Blood and Marrow Transplant Research (CIBMTR) confirmed these results in a cohort of 1514 patients transplanted for MDS [71]. In this cohort, 289 patients had *TP53* mutations. In a multivariate analysis, *TP53* mutations were associated with shorter survival and a shorter time to relapse. The 3 years OS was 20% and the median OS was 0.7 years. In this study, a *TP53* variant allele fraction (VAF) of 10% or higher was not significantly associated with survival and neither was the presence of multiple *TP53* mutations. Interestingly, another study found the mutation type had an impact on transplant outcome. Indeed, patients with only truncating mutations had shorter survival than patients exhibiting missense mutations. Another study identified the *TP53* allelic state as an independent prognostic factor for *TP53*-mutated AML patients following aHSCT [12]. In this retrospective cohort analysis of 36 patients with *TP53*, the authors showed a trend for longer overall survival in monoallelic patients as compared to multi-hit patients following HSCT. These results emphasized the importance of analyzing the *TP53* state in future transplantation studies. In an attempt to identify different prognostic factors in *TP53*-mutated AML transplanted patients, biological samples of a cohort of 83 consecutive patients transplanted for *TP53* AML or MDS at a single center were analyzed between February 2011 and March 2017 [72]. The median age was 60 years. In this study, 71% of the patients received a myeloablative conditioning, and 59% of the grafts were peripheral blood stem cells. Of note, 16% of the patients received subcutaneous azacitidine maintenance following HSCT. A multivariate analysis showed the melphalan-based regimen (hazard ratio [HR], 6.5; 95% CI, 2.1–20; *p* = 0.001), Karnofski Performans Status (KPS) ≤80% (HR, 2.8; 95% CI, 0.96–8; *p* = 0.06), and treatment-related AML/MDS (HR, 4.2; 95% CI, 1.5–12; *p* = 0.007). Regarding overall survival predictive factors, HCT-CI > 4 (HR, 3.9; 95% CI, 1.2–13; *p* = 0.03), KPS ≤80% (HR, 3.04; 95% CI, 1.6–5.9; *p* = 0.001), and disease not in CR1/2 (HR, 4.1; 95% CI, 1.5–11; *p* = 0.004) were associated with worse OS. In this study, azacitidine maintenance did not affect the patient’s outcome. The study also reports better progression-free survival for patients receiving higher doses of busulfan, emphasizing the need to reduce the leukemic-cell burden before transplant.

It has been previously shown that MRD negativity at transplant correlates with a better post-transplant outcome [73]. A retrospective analysis from the Moffitt Cancer Center on 47 patients who received an aHSCT for *TP53* mut MDS/AML, reported that patients who achieved a clearance of the *TP53* mutation (NGS) at the time of aHSCT after receiving HMA treatment, had a significantly better OS than those who did not achieve clearance (median OS of 21.73 months vs. 6.44 months, *p* = 0.042) [74]. In the pre-transplant setting, the addition of eprenetapopt to demethylating agents offers more efficient treatment options to these usually chemoresistant AML subsets. As presented above, in a phase Ib/II trial, eprenetapopt combined with azacitidine yielded complete remission, including complete molecular remission [41]. In this study 40% of the patients proceeded to aHSCT. The median time to aHSCT was 5.6 months (range, 1.7–9.7 months). The good complete response rate did not translate into an improved survival rate after aHSCT, as the median OS for patients who were bridged to aHSCT was 14.7 months (95% CI, 8.6 to 20.9). Moreover, in this study, aHSCT was not significantly associated with survival (hazard ratio, 1.01; *p* = 0.98). However, a subgroups analysis revealed several factors that may influence the outcome of *TP53* AML patients treated with a combination of azacitidine and eprenetapopt. The number of cycles patients received before transplant impacted aHSCT survival. Indeed, OS was significantly better in the group of patients receiving at least four cycles of combination therapy prior to aHSCT compared with those who received less (16.1 months; 95% CI, 10.4 to not reported [NR] v 9.3 months; 95% CI, 8 to NR months, respectively; *p* = 0.01). 

In the post-transplantation setting, the possibility of post-transplantation maintenance with eprenetapopt in combination with azacytidine for *TP53* AML patients has been assessed in a phase 2 clinical trial (NCT03931291) [75]. In a cohort of 33 patients who received aHSCT for AML/MDS with *TP53* mutations, 1-year relapse-free survival was 58% with an OS at 76%. Median RFS and OS were 12.1 and 19.3 months, respectively. These truly encouraging results confirm the necessity of post transplantation intervention for *TP53*-mutated AML and MDS. 

Another factor accounting for the high relapse incidence following aHSCT is the highly immunosuppressive microenvironment surrounding the *TP53*-mutant leukemic cell. Among the immune escape mechanisms is the expression of inhibitory PDL1 [76]. A recent report demonstrated a significant increase of PDL1 expression at the surface of hematopoietic stem cells of patients with *TP53* mutations [77]. *TP53*-mutated AML patients also exhibited reduced numbers of bone marrow–infiltrating OX40 + cytotoxic T cells and helper T cells. Further adding to the immunosuppressive characteristics of the microenvironment was the increased frequency of highly suppressive Tregs. A donor lymphocyte infusion is one of the available options to bypass T-cell exhaustion [78]. 

A better understanding of the mechanisms underlying the immune escape of *TP53*-mutated AML is essential to improve the outcome for these patients following aHSCT. A greater response before transplantation, combined with increased alloreactivity post transplantation using donor lymphocyte infusions with or without chemical maintenance with eprenetapopt, sabatolimab, azacytidine, or venetoclax are some of the options currently under investigation.

## 9. Conclusions

*TP53*-mutated AML and MDS are some of the myeloid malignancies with the poorest prognosis. The prognoses are heterogeneous, depending on mono or biallelic mutations. The emergence of new drugs with several mechanisms of action are encouraging. These new drugs induce a greater response and may cure some patients using aHSCT. Several sequential strategies need to be evaluated to identify the best therapeutic strategy to cure these patients.

## Figures and Tables

**Table 1:** Outcome of *TP53*-mutated myeloid malignancies patients treated with intensive chemotherapy. CR: Complete Remission, EFS: Event Free Survival, OS: Overall Survival.

Regimen	Demographics	CR	EFS	OS	References
Cytarabine + anthracycline (7 + 3) and CPX 351	18–85 y/o	28–48%	3 years EFS 1–6%	3 years OS 3–8%	[16,18,19,20,21,22,23,24,25,26,27,28,29,30,31,32,33]

**Table 2:** Outcome of *TP53*-mutated myeloid malignancy patients treated with hypomethymating agents. CR: Complete Remission, EFS: Event Free Survival, OS: Overall Survival.

Regimen	Demographics	CR	EFS	OS	References
Azacitidine	20–91 y/o	20–40%	Not available	Median OS: 7 months	[34,35,36,41]
Decitabine	47–90 y/o	30%	Median EFS: 6 months	Median OS: 2.1–7 months	[28,35,37,38,39,40]

**Table 3:** Outcome of *TP53*-mutated myeloid malignancy patients treated with a combination of hypomethymating agents. CR: Complete Remission, CRi: Complete Remission with Incomplete count recovery, EFS: Event Free Survival, OS: Overall Survival.

Regimen	Demographics	CR/CRi	EFS	OS	References
Azacitidine + venetoclax OR Decitabine + venetoclax	22–86 y/o	43–67%	Median EFS: 6–7 months	Median OS: 5–7 months	[42,43,44,45,46]
Azacitidine + eprenetapopt	34–87 y/o	44%	Not available	Median OS: 11 months	[47,48]
Azacitidine + magrolimab	Median age 72 y/o	45% CR, 14% CRi	Not available	Median OS: 12.9 months	[49]

**Table 4:** Outcome of *TP53*-mutated myeloid malignancy patients after allogeneic stem cell transplantation. AML: Acute Myeloid Leukemia, CR: Complete Remission, EFS: Event Free Survival, MAC: Myeloablative Conditioning, MDS, Myelodysplastic Syndrome, OS: Overall Survival, PFS: Progression Free Survival, RIC: Reduced Intensity Conditioning.

Pathology	N	Conditioning	RFS	OS	NRM	Reference
AML (62%)/MDS (38%) with 17p abnormalities	30	RIC (63%)/MAC (37%)	Median RFS: 6 months (AML)–4 months (MDS)	Median OS: 18 months (AML)–11 months (MDS)	9.1% (MDS)–10.6% (AML)	[67]
AML with 17p abnormalities	139	RIC (59%)/MAC (41%)	2 years LFS: 24%	2 years OS: 28%	2 years NRM: 15%	[68]
*TP53* mut MDS	289	RIC/MAC	Not available	3 years OS: 20% Median OS: 8 months	2 years NRM: 35%	[69]
*TP53* mut AML/MDS	83	RIC (29%)/MAC (79%)	1 year PFS: 25% Median PFS: 5 months	1 year OS: 35% Median OS: 8 months	1 year NRM: 20%	[70]
